# Combustion smoke-induced inflammation in the olfactory bulb of adult rats

**DOI:** 10.1186/s12974-014-0176-5

**Published:** 2014-10-10

**Authors:** Ying-Ying Zou, Yun Yuan, Enci Mary Kan, Jia Lu, Eng-Ang Ling

**Affiliations:** Department of Pathology and Pathophysiology, Faculty of Basic Medical Sciences, Kunming Medical University, 1168 West Chunrong Road, Kunming, 650500 PR China; Department of Anatomy and Histology/Embryology, Faculty of Basic Medical Sciences, Kunming Medical University, 1168 West Chunrong Road, Kunming, 650500 PR China; Defense Medical and Environmental Research Institute, DSO National Laboratories, 27 Medical Drive, Singapore, 117510 Singapore; Department of Anatomy, Yong Loo Lin School of Medicine, National University of Singapore, 4 Medical Drive, MD10, Singapore, 117597 Singapore

**Keywords:** edema, inflammation, nitric oxide synthase, olfactory bulb, smoke

## Abstract

**Background:**

The damaging effect of combustion smoke inhalation on the lung is widely reported but information on its effects on the olfactory bulb is lacking. This study sought to determine the effects of smoke inhalation on the olfactory bulb, whose afferent input neurons in the nasal mucosa are directly exposed to external stimuli, such as smoke.

**Methods:**

Adult male Sprague-Dawley rats were subjected to combustion smoke inhalation and sacrificed at different time points. Changes in olfactory bulb proteins including vascular endothelial growth factor (VEGF), inducible nitric oxide synthase (iNOS), endothelial nitric oxide synthase (eNOS), neuronal nitric oxide synthase (nNOS), Na^+^-K^+^-Cl^−^ cotransporter 1 (NKCC1), glial fibrillary acidic protein (GFAP), and aquaporin-4 (AQP4) were evaluated by Western blot analysis. In addition, ELISA was conducted for cytokine and chemokine levels, and double immunofluorescence labeling was carried out for GFAP/VEGF, GFAP/AQP4, NeuN/nNOS, GFAP/NKCC1, NeuN/NKCC1, GFAP/Rhodamine isothiocyanate (RITC), and transferase dUTP nick end labeling (TUNEL). Aminoguanidine was administered to determine the effects of iNOS inhibition on the targets probed after smoke inhalation.

**Results:**

The results showed a significant increase in VEGF, iNOS, eNOS, nNOS, NKCC1, and GFAP expression in the bulb tissues, with corresponding increases in inflammatory cytokines and chemokines after smoke inhalation. Concurrent to this was a drastic increase in AQP4 expression and RITC permeability. Aminoguanidine administration decreased the expression of iNOS and RITC extravasation after smoke inhalation. This was coupled with a significant reduction in incidence of TUNEL + cells that was not altered with administration of L-NG-nitroarginine methyl ester (L-NAME).

**Conclusions:**

These findings suggest that the upregulation of iNOS in response to smoke inhalation plays a major role in the olfactory bulb inflammatory pathophysiology, along with a concomitant increase in pro-inflammatory molecules, vascular permeability, and edema. Overall, these findings indicate that the olfactory bulb is vulnerable to smoke inhalation.

## Background

Smell is a major sensory function and plays an important role in identification, alarm, appetite, and mood [1]; it is mediated by the olfactory bulb. The olfactory bulb participates in many physiological functions, such as appetite regulation, lactation, response to adverse environments, and social interaction [2,3]. It contains a large number of afferent input sensory cells that might be directly exposed to undesirable external stimuli, such as smoke; hence, damage to the olfactory bulb from smoke inhalation could affect the noted functions.

Smoke inhalation injury occurs from the inhalation of the by-products of combustion, which includes toxic gases, such as carbon monoxide and cyanide, and particulate matter [4-6]. The damaging effects of smoke inhalation on the pulmonary system are widely reported, with most of the current literature focused on the management of acute smoke inhalation-induced damage on the respiratory system [7]. Smoke inhalation injury has been reported to lead to a 20% increase in mortality in burn patients. In conjunction with such comorbidities as pneumonia, mortality can further increase by up to 60% [8]. The effects of smoke inhalation on the central nervous system are less well researched, and indeed, even more so regarding the olfactory bulb [9-13].

We reported previously that the acute inhalation of combustion smoke caused pathologic changes in the adult rat retina, cerebellum, and hippocampus, which were attributed to increased vascular permeability and edema [12,13]. Here we have extended our study by investigating changes in the olfactory bulb of the adult rat subjected to a similar smoke challenge. Combustion smoke is known to result in a hypoxic environment consisting of elevated carbon monoxide and decreased oxygen. So far, there have been limited studies reporting injury to the olfactory bulb as a result of hypoxia [14-16]. As far as can be ascertained, there is no available literature on the effects of combustion smoke inhalation on the olfactory bulb. Therefore, this study was designed to determine whether the neurons or glial cells of the olfactory bulb would also be affected by combustion smoke inhalation.

In view of the fact that inflammatory mediators, such as TNF-α, interleukin-1 β (IL-1β), glutamate, nitric oxide synthase (NOS), vascular endothelial growth factor (VEGF), glial fibrillary acidic protein (GFAP), Na^+^-K^+^-Cl^−^ cotransporter 1 (NKCC1), and aquaporin-4 (AQP4), are altered in brain injuries [13,17-19], it was surmised that these molecular biomarkers in the olfactory bulb might also be affected following combustion smoke inhalation. Hence, we sought to determine whether these protein markers in the olfactory bulb would be altered as a result of smoke inhalation.

## Methods

### Animals

A total of 155 adult male Sprague-Dawley rats (body weight 280 to 320 g, ages 6 to 8 weeks) were used. All experiments were carried out in accordance with the National Institute of Health Guide for the Care and Use of Laboratory Animals (NIH Publications No. 80-23). The project was approved by the Institutional Animal Care and Use Committee (IACUC), DSO National Laboratories (Protocol Number: 09/87) and the National University of Singapore (Protocol 088/07). All efforts were made to minimize the number of rats used, as well as stress and suffering. The rats were randomly divided into normal control (C), smoke inhalation plus saline (SI + S), smoke inhalation + aminoguanidine (SI + AG) and smoke inhalation + NG nitro L arginine methyl ester (SI + L-NAME) groups. Table 1 shows the number of animals used for smoke challenge, drug intervention, and the various tests investigated.

**Table 1:** **Number of rats used in different experiments**

	**Control**	**0.5 h**	**3 h**	**24 h**	**72 h**	**Day 7**	**Day 14**	**Total**
Inflammatory cytokines assay	3			SI + S 3				9
				SI + AG 3				
Western blotting	4	SI + S 4	+ S 4	SI + S 4	SI + S 4			28
				SI + AG 4	SI + AG 4			
Double immunofluorescence	4			SI + S 4	SI + S 4	+ S 4	+ S 4	28
				SI + AG 4	SI + AG 4			
Nitrite assay	4	SI + S 4	SI + S 4	SI + S 4	SI + S 4			44
				SI + AG 4	SI + AG 4			
			SI + AG 4	SI + N 4	SI + N 4			
			SI + N 4					
Glutamate assay	4	SI + S 4	SI + S 4	SI + S 4	SI + S 4			28
				SI + AG 4	SI + AG 4			
Assessment of vascular permeability	3			SI + S 3				9
				SI + AG 3				
Detection of apoptosis	3				SI + S 3			9
					SI + AG 3			
Total								155

SI + AG, smoke inhalation plus aminoguanidine; SI + N, smoke inhalation plus L-NAME; SI + S, smoke inhalation plus saline.

### Smoke challenge

The entire smoke inhalation testing set-up was designed with reference to Lee *et al*. (2005) [9] and Whitehead *et al.* (2003**)** [6] and was adopted by us previously [12]. Briefly, the revised set-up was designed and built to achieve a constant smoke challenge of constant smoke toxicants from burning cotton. The set-up consists of three main parts: a furnace, an equilibrium chamber to contain the volume of smoke being generated by the furnace, and animal exposure chambers. Smoke was generated by burning 33 g of cotton towel in a 290°C furnace for 5 min and then allowed to collect and cool in the equilibrium chamber. Unanesthetized awake animals were placed individually in up to two exposure chambers and allowed to acclimatize for 15 min before release of the smoke from the equilibrium chamber. The animals were exposed to the smoke mixture for 60 min. Gas concentration was monitored by a carbon monoxide and oxygen combustion meter (Testo AG, Lenz-kirch, Germany). In this experimental paradigm, we have established that the following smoke challenge conditions would result in an approximate mortality rate of 10%: CO level, 2,200 to 2,500 ppm; O_2_ level, >19%. Fresh air was allowed to recirculate into the test chambers if either the CO level reached over 2,500 ppm or the O_2_ level dropped below 19%, to prevent death from hypoxia or CO-poisoning. Normal control rats were not subjected to smoke inhalation.

### Drug administration

The rats were given intraperitoneal injections of aminoguanidine (100 mg/kg of body weight; Sigma, St Louis, MO, USA) [20] or L-NAME (300 mg/kg of body weight; Sigma) [21], with the first injection given immediately after smoke inhalation, followed by an injection every 24 h until the respective harvest time points. The SI + S group received an intraperitoneal injection of an equal volume of saline after smoke inhalation. Fresh olfactory bulbs were removed at the time of sacrifice and prepared for Western blotting analysis and measurement of nitric oxide production. In parallel to this, another group of rats was examined for vascular permeability changes in the olfactory bulb following SI + S and SI + AG.

### Inflammatory cytokine assay

The relative protein concentrations of 12 pro-inflammatory cytokines to control samples in the protein supernatant from the olfactory bulb tissue lysate of rats subjected to combustion smoke inhalation (*n* =3 at 24 h after smoke inhalation) were determined with a Rat Inflammatory Cytokines Multi-Analyte ELISArray kit (Mer004A; Qiagen, Valencia, CA, USA). The tissue homogenates for the ELISArray measurements were prepared as for Western blotting and ELISArray measurements were performed according to the manufacturer’s protocol.

### Western blotting analysis

At designated time points after smoke inhalation, the rats were anesthetized with ketamine (75 mg/kg) and xylazine (10 mg/kg) intraperitoneally and were sacrificed by cardiac puncture. After sacrifice, fresh olfactory bulb tissue from the C (*n* =4), SI + S (*n* =4 each at 0.5, 3, 24, and 72 h after smoke inhalation) and SI + AG (*n* =4 each at 24 and 72 h after smoke inhalation) groups were removed and were snap-frozen in liquid nitrogen and stored at −80°C. The olfactory bulb tissue proteins were extracted using a protein extraction kit (Pierce Biotechnology, Inc., Rockford, IL, USA) containing protease inhibitors. All procedures were carried out at 4°C. Homogenates were centrifuged at 15,000 *g* for 15 min and the supernatant collected. The protein concentration of the samples was determined by the Bradford method using BSA (Bio-Rad Laboratories, Hercules, CA, USA). Samples of supernatants containing 35 mg of protein were heated to 95°C for 5 min and were separated on 8% sodium dodecyl sulphate–polyacrylamide gels (for inducible NOS (iNOS), neuronal NOS (nNOS), endothelial NOS (eNOS), and NKCC1), and 12% sodium dodecyl sulphate-polyacrylamide gels (for GFAP, VEGF, and AQP4) using a Mini Protein II apparatus (Bio-Rad Laboratories). Protein bands were electroblotted onto 0.45 mm polyvinylidene difluoride membranes (Bio-Rad Laboratories) using a semidry electrophoretic transfer cell. The membranes were washed with Tris-buffered saline (TBS)–0.1% Tween buffer and then blocked with 5% w/v non-fat dry skim milk for 60 min at room temperature. After this, they were incubated with primary antibodies (Table 2) in blocking solution overnight on a shaker at 4°C. After rinsing with TBS-0.1% Tween, the membranes were incubated with horseradish peroxidase-conjugated secondary antibody (1:2,000 to 10,000; Pierce Biotechnology, Inc.) for 1 h. Proteins were revealed by an enhanced-chemiluminescence detection system according to the manufacturer’s instruction (Super Signal West Pico Horseradish Peroxidase detection kit; Pierce Biotechnology, Inc.) and developed on film. The band intensity of target protein levels relative to the housekeeping protein, β-actin, was quantified using the scanning densitometer and Quantity One Software, version 4.4.1 (Bio-Rad Laboratories).

**Table 2:** **Primary antibodies used in Western blotting analysis**

**Name**	**Dilution**	**Source**	**Catalog number**
AQP4	1:2,500	Santa Cruz Biotechnology, Inc., CA, USA	SC-20978
VEGF	1:500	Santa Cruz Biotechnology, Inc. CA, USA	SC-7269
iNOS	1:2,500	BD Biosciences, CA, USA	610329
nNOS	1:500	BD Biosciences, CA, USA	610311
eNOS	1:500	BD Biosciences, CA, USA	610296
GFAP	1:1,000	Millipore Corporation, Bioscience, Billerica, MA, USA	MAB360
NKCC1	1:500	Millipore Corporation, Bioscience, Billerica, MA, USA	AB3560P
β-actin	1:10,000	Sigma, MO, USA	A1978

AQP4, aquaporin-4; eNOS, endothelial nitric oxide synthase; GFAP, glial fibrillary acidic protein; iNOS, inducible nitric oxide synthase; NKCC1, Na^+^-K^+^-Cl^−^ cotransporter 1; nNOS, neuronal nitric oxide synthase; VEGF, vascular endothelial growth factor.

### Double immunofluorescence

The C (*n* =4), SI + S (*n* =4 each at 24, 72 h, 7 days, and 2 weeks after smoke inhalation) and SI + AG (*n* =4 each at 24 and 72 h after smoke inhalation) groups were used for immunofluorescence studies. These time points were chosen because Western blotting analysis showed obvious changes in the inflammatory targets of interest in the levels following smoke inhalation over this period*.* At the designated time points, the rats were anesthetized with intraperitoneal ketamine (75 mg/kg) and xylazine (10 mg/kg) and then perfused transcardially with saline, followed by 2% paraformaldehyde in 0.1 M phosphate buffer. The olfactory bulbs were removed, post-fixed for 2 h in the same fixative, then cryoprotected in 15% sucrose for 24 to 48 h. Frozen coronal sections, 35 μm thick, were cut with a cryostat (Leica CM 3050), mounted onto gelatin-coated microscopic slides, and stored at −20°C until use. Tissue sections at different time points were incubated in primary antibodies (Table 3). After incubation, FITC-conjugated and Cy3-conjugated secondary antibodies were added. Images representing at least one brain bulb each from four rats at different time points were captured under a confocal microscope (Olympus Fluoview TM1000). Immunofluorescence labeling for the various antibodies directed against the respective cell types was consistent and reproducible across different rats.

**Table 3:** **Primary antibodies used in immunofluorescence studies**

**Name**	**Dilution**	**Source**	**Catalog number**
AQP4	1:250	Santa Cruz Biotechnology, Inc., CA, USA	SC-20978
VEGF	1:100	Santa Cruz Biotechnology, Inc. CA, USA	222-P
GFAP	1:500	Millipore Corporation, Bioscience, Billerica, MA, USA	MAB360
NKCC1	1:200	Millipore Corporation, Bioscience, Billerica, MA, USA	AB3560P
nNOS	1:200	BD Biosciences, CA, USA	610311
NeuN	1:500	Abcam, Cambridge, MA, USA	ab177487

AQP4, aquaporin-4; GFAP, glial fibrillary acidic protein; NKCC1, Na^+^-K^+^-Cl^−^ cotransporter 1; nNOS, neuronal nitric oxide synthase; VEGF, vascular endothelial growth factor.

### Nitrite assay

The total amount of NO (μM) in the olfactory bulb samples from the C (*n* =4), SI + S (*n* =4 each at 0.5, 3, 24, and 72 h after smoke inhalation), SI + AG, and SI + L-NAME (*n* =4 each at 3, 24, and 72 h after smoke inhalation) groups was assessed by the Griess reaction, using a colorimetric assay kit (US Biological, Swampscott, MA, USA) that detects nitrite. Homogenates and supernatant, as described for Western blotting, were prepared for NO^2−^ measurements. Briefly, 85 μl of each sample was added to 5 μl of nitrate reductase followed by 5 μl enzyme cofactor according to the manufacturer’s instructions and incubated for 1 h at room temperature. Next, 5 μl of enhancer was added and this was incubated for 10 min. Following this, 50 μl of Griess reagent R1 and 50 μl of Griess reagent R2 were added to the solution and the color was allowed to develop at room temperature for 10 min. The optical density of the samples was measured at 540 nm using the GENios microplate reader (Tecan Austria GmbH, Salzburg, Austria). Nitrite concentration was determined using a nitrite standard curve.

### Glutamate assay

The glutamate concentrations (mg/ml) in the olfactory bulb protein supernatant from the C (*n* =4), SI + S (*n* =4 each at 0.5, 3, 24, and 72 h after smoke inhalation) and SI + AG (*n* =4 each at 24 and 72 h after smoke inhalation) groups were determined using a Glutamate BioAssay kit (US Biological, Swampscott, MA, USA; catalog number G7114). The protein lysates were prepared as for Western blotting and the bioassay measurements were performed according to the manufacturer’s protocol.

### Assessment of vascular permeability

Rhodamine isothiocyanate (RITC, molecular weight = 536.08) **(**Sigma, MO, USA**)** was used as a fluorescent tracer to evaluate vascular permeability. The C, SI + S, and SI + AG groups (*n* =3 per group) were given an intraperitoneal injection of RITC (5 μl of 1% RITC/g body weight) dissolved in normal saline. The RITC was administered to rats 24 h after smoke inhalation. This time point was chosen because immunofluorescence and Western blot analysis showed obvious changes in the olfactory bulb following smoke inhalation over this period. The rats were sacrificed at 3 h after RITC administration. Following perfusion with 2% paraformaldehyde in 0.1 M phosphate buffer, the olfactory bulbs were removed and frozen coronal sections, 35 μm thick, were cut and processed, as described previously. Immunofluorescence labeling was carried out using GFAP antibody (Millipore Corporation, Bioscience, Billerica, Massachusetts, USA), following the same method as for double immunofluorescence labeling.

### Detection of apoptosis

To detect cells undergoing apoptosis in the olfactory bulb, frozen sections as for double immunofluorescence labeling were derived from the C (*n* =3), SI + S (*n* =3 at 72 h after smoke inhalation), and SI + AG (*n* =3 at 72 h after smoke inhalation) groups were assessed using a terminal deoxynucleotidyl transferase dUTP nick end labeling (TUNEL) apoptosis detection kit (Millipore Corporation, Bioscience, Billerica, Massachusetts*,* USA). The frozen sections were fixed with 4% paraformaldehyde in 0.1 M phosphate buffer, pH 7.4, and subsequently washed with three lots of PBS for 10 min, permeabilized with 0.2% Triton-X100 in PBS twice for 10 min at room temperature, and subsequently washed three times with PBS for 5 min at 37°C. The remaining steps were performed according to the manufacturer’s instructions. The sections were counterstained with 4',6-diamidino-2-phenylindole (DAPI), washed with PBS, and mounted with a fluorescent mounting medium (Dako Cytomation, Glostrup, Denmark). TUNEL-positive cells were enumerated by counting the labeled cells in eight randomly selected microscopic fields obtained from each specimen at 40× and 80× magnification. The percentage of TUNEL-positive cells was calculated and averaged.

### Statistical analysis

For Western blots and multi-analyte ELISArray, data are reported as mean ± standard deviation. Data were analyzed using one-way ANOVA followed by post-hoc analysis using Dunnet’s test (SPSS version 15.0 software, Chicago, IL, USA) to determine the statistical significance of differences between the C, SI + S, SI + AG, and SI + L-NAME groups. All experiments were conducted in triplicate from different tissue samples. Significance is accepted as *P* <0.05 and is denoted by an asterisk (* *P* <0.05; ** *P* <0.01).

## Results

### Normal histology of the olfactory bulb

**Figure 1:**
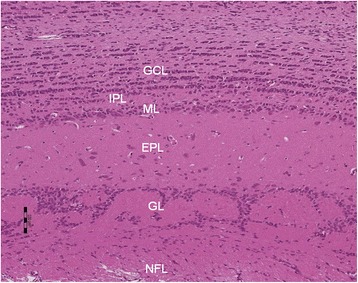
**H & E staining of normal olfactory bulb tissue.** The olfactory bulb shows six well delineated layers, namely, the nerve fiber layer (NFL), glomerular layer (GL), external plexiform layer (EPL), mitral cell layer (ML), internal plexiform layer (IPL), and granule cell layer (GCL). Scale bar =100.0 μm.

With H & E staining, the olfactory bulb showed six well delineated layers, namely, the nerve fiber layer, glomerular layer, external plexiform layer, mitral cell layer, internal plexiform layer, and granule cell layer (Figure 1). In response to blood gas changes, as previously reported [12], reactive alterations were observed affecting glial reactions and expression of cytokines and chemokines in bulb tissue protein extracted *in toto*. The following immunofluorescence observations were focused mainly on reactive changes due to smoke inhalation in the external plexiform layer, mitral cell layer, and internal plexiform layer.

### Elevated inflammatory cytokines after smoke inhalation

**Figure 2:**
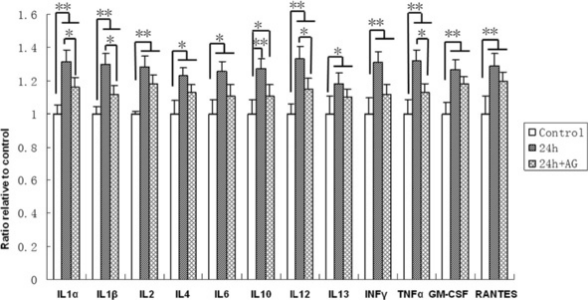
**Elevated cytokines and chemokines.** The concentration levels of the various cytokines IL-1α, IL-1β, IL-2, IL-4, IL-6, IL-10, IL-12, IL-13, IFN-γ, TNF-α, GM-CSF, and RANTES are increased significantly at 24 h in the SI + S group, as compared with matched controls. The increase was most substantial for IL-12, IFN-γ and TNF-α in the SI + S group, compared with the matched control. In the SI + AG group, the levels of IL-1α, IL-1β, IL-12, and TNF-α were significantly decreased compared with the SI + S group. * *P* <0.05; ** *P* <0.01.

In the olfactory bulb tissue, the concentration levels of the cytokines IL-1α, IL-1β, IL-2, IL-4, IL-6, IL-10, IL-12, IL-13, IFN-γ, TNF-α, GM-CSF, and RANTES were increased significantly at 24 h in the SI + S group when compared with the matched control (Figure 2). In particular, the increase was most significant (*P* <0.01) for IL-12, IFN-γ, and TNF-α in the SI + S group, as compared with the matched control. Administration of aminoguanidine significantly decreased the expression levels of IL-1α, IL-1β, IL-12, and TNF-α, compared with the SI + S group at 24 h (Figure 2).

### Western blotting analysis

**Figure 3:**
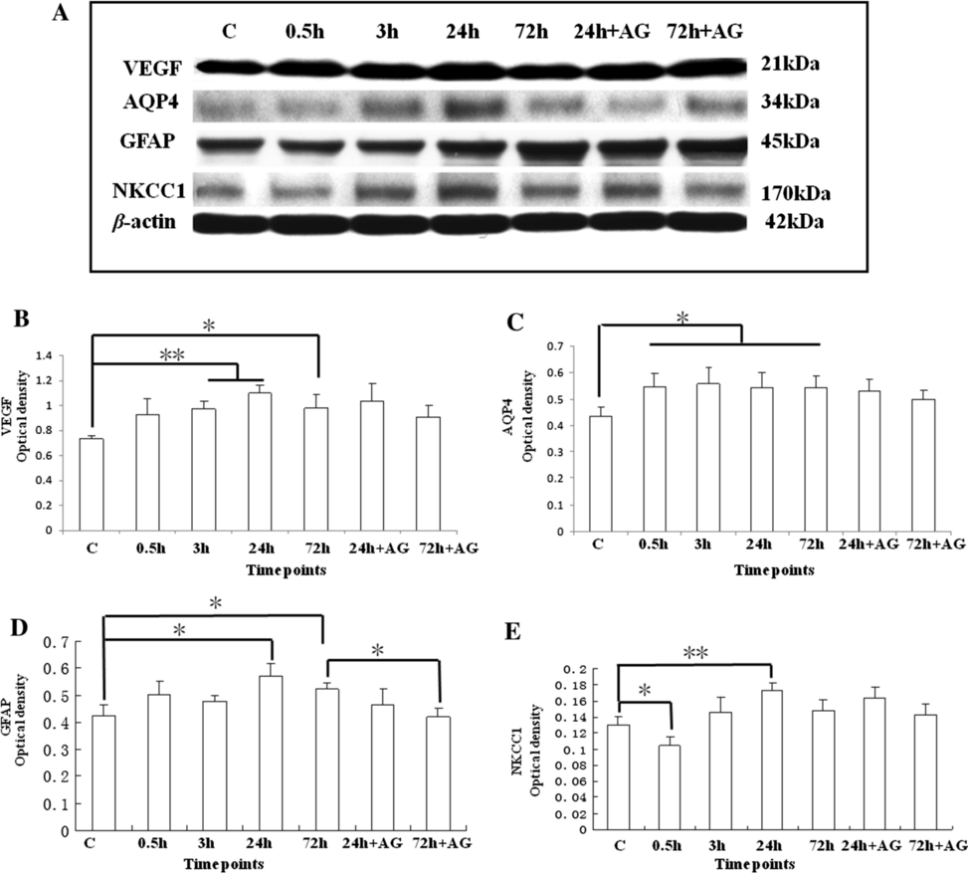
**Western blotting analysis of VEGF, AQP4, GFAP, and NKCC1 protein expression levels in the olfactory bulb of the C group (lane 1); SI + S group, at 0.5 h (lane 2), 3 h (lane 3), 24 h (lane 4), and 72 h (lane 5); and SI + AG group, at 24 h (lane 6) and 72 h (lane 7). (A)** VEGF (21 kDa), AQP4 (34 kDa), GFAP (45 kDa), NKCC1 (170 kDa), and β-actin (42 kDa) immunoreactive bands. Bar graphs represent optical density (mean ± standard deviation) of **(B)** VEGF, **(C)** AQP4, **(D)** GFAP, and **(E)** NKCC1 normalized to β-actin for C, SI + S at 0.5, 3, 24, and 72 h and SI + AG at 24 h and 72 h (* *P* <0.05; **P <0.01). (B) VEGF shows a steady increase in the SI + S group at 0.5, 3, 24, and 72 h, as compared with that of the control with recovery at 72 h in the SI + AG group, as compared with 72 h. (C) AQP4 shows an acute increase at 0.5, 3, 24, and 72 h in the SI + S group, as compared with the control. (D) GFAP shows a significant increase at 24 and 72 h in the SI + S group, as compared with the control, with recovery at 72 h in the SI + AG group, as compared with 72 h. (E) NKCC1 shows a decrease at 0.5 h but rises after this peaking at 24 h in the SI + S group, as compared with the control. (* *P* <0.05, ** *P* <0.01).

**Figure 4:**
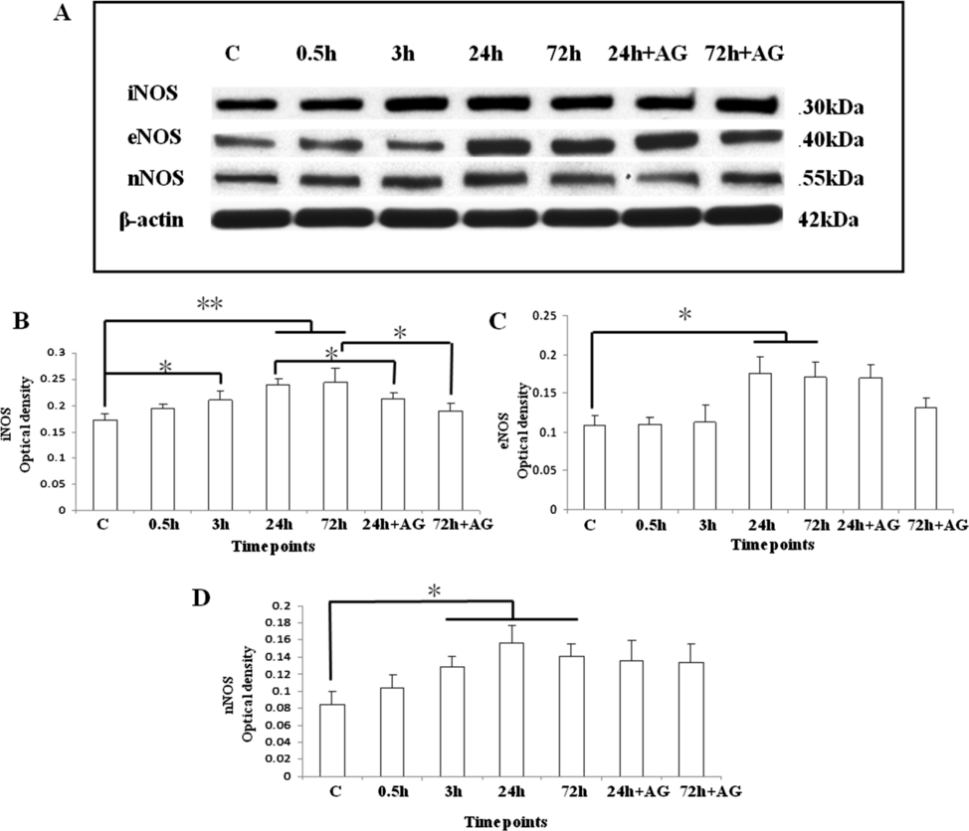
**Western blotting analysis of iNOS, eNOS, and nNOS protein expression levels in the olfactory bulb of the C group (lane 1); the SI + S group at 0.5 h (lane 2), 3 h (lane 3), 24 h (lane 4) and 72 h (lane 5); and the SI + AG group at 24 h (lane 6) and 72 h (lane 7). (A)** iNOS (130 kDa), eNOS (140 kDa), nNOS (155 kDa), and β-actin (42 kDa) immunoreactive bands. Bar graphs represent optical density (mean ± standard deviation) of **(B)** iNOS, **(C)** eNOS, and **(D)** nNOS normalized to β-actin for C, SI + S at 0.5, 3, 24, and 72 h and SI + AG at 24 and 72 h (* *P* <0.05; ** *P* <0.01). **(B)** iNOS shows a significant increase in the SI + S group at 3, 24, and 72 h, as compared with the C group, with recovery at 72 h in the SI + AG group, as compared with the SI + S group at 72 h. **(C)** eNOS shows an increase in the SI + S group at 24 and 72 h, as compared with the C group. Administration of aminoguanidine did not affect its expression level significantly. **(D)** nNOS shows a significant increase in the SI + S group at 3 and 24 h, as compared with the control. Aminoguanidine administration did not affect its expression level significantly.

By Western blotting analysis, the olfactory bulb tissue showed a progressive and significant increase in protein levels of VEGF, AQP4, GFAP, NKCC1, iNOS, eNOS, and nNOS over time in SI + S group (Figures 3 and 4). Densitometry analysis of the VEGF-immunoreactive band of approximately 21 kDa showed a significant increase at 3 h (*P* <0.01), 24 h (P*p* <0.01), and 72 h (*P* <0.05) in the SI + S group, as compared with that of the C group (Figure 3A,B). In the SI + AG group, the VEGF protein expression level remained relatively unaltered at 24 and 72 h compared with the SI + S group (without aminoguanidine treatment) (Figure 3A,B).

AQP4 was detected as a major band at approximately 34 kDa, and showed a significant increase in optical density in the SI + S group at 0.5, 3, 24, and 72 h (*P* <0.05) in comparison with control levels (Figure 3A,C). Administration of aminoguanidine (SI + AG) did not affect AQP4 optical density compared with the SI + S group at 24 and 72 h.

GFAP was detected as a major band at approximately 45 kDa. In the SI + S group, GFAP protein expression level was significantly increased at 24 and 72 h (*P* <0.05), compared with the C group (Figure 3A,D). In the SI + AG group, GFAP protein expression level was significantly decreased at 72 h, compared with the SI + S group (Figure 3A,D).

NKCC1 was detected as a major band at approximately 170 kDa. In the SI + S group, the NKCC1 protein expression level showed a substantial increase at 24 h (*P* <0.01), compared with the C group (Figure 3A,E). The NKCC1 protein expression level in the SI + S group appeared to decline at 0.5 h, compared with the C group (Figure 3A,E). Aminoguanidine administration did not affect the expression level of NKCC1.

The iNOS immunoreactive bands, with a molecular weight of approximately 130 kDa, increased significantly in optical density from 3 to 72 h (*P* <0.05, *P* <0.01, *P* <0.01, respectively) in the SI + S group, compared with the C group levels (Figure 4A,B). In the SI + AG group at 24 and 72 h, iNOS protein expression level was decreased significantly (*P* <0.05), as compared with the SI + S group at the same time points (Figure 4A,B).

The eNOS immunoreactive bands, with a molecular weight of approximately 140 kDa, increased significantly in optical density in the SI + S group at 24 and 72 h (*P* <0.05) compared with the C group levels (Figure 4A,C). Expression of eNOS was not altered by aminoguanidine treatment.

The nNOS-immunoreactive bands, with a molecular weight of approximately 155 kDa, showed a significant increase in optical density in the SI + S group at 24 and 72 h (*P* <0.05) in comparison with the C group levels (Figure 4A,D). At 72 h in the SI + AG group, the nNOS protein expression level remained elevated (Figure 4A,D).

### Double immunofluorescence labeling

#### Expression of VEGF and GFAP

**Figure 5:**
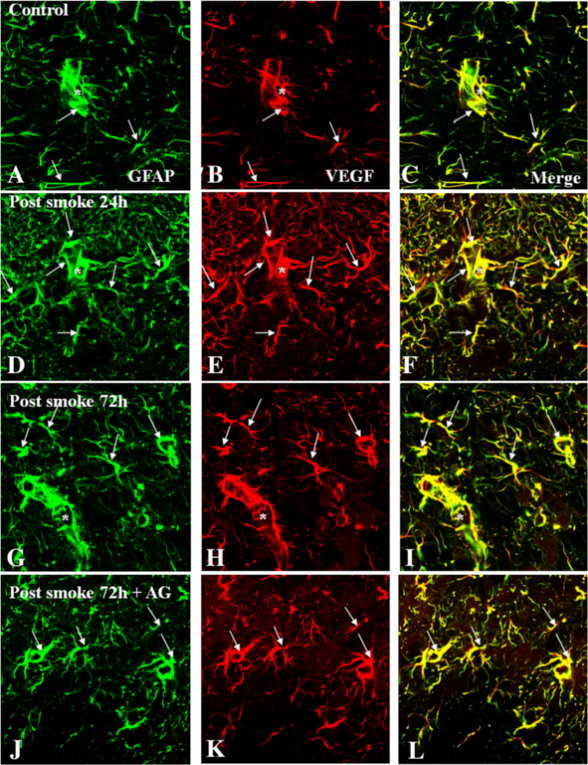
**VEGF (red) and GFAP (green) immunopositive astrocytes in the external plexiform layer, mitral cell layer, and internal plexiform layer in the olfactory bulb of the C group (A-C), SI + S group at 24 and 72 h (D-I), and SI + AG group at 72 h (J-L).** In the control, moderate VEGF immunofluorescence is detected in GFAP-labeled astrocytes. In the SI + S group, VEGF immunofluorescence is evidently enhanced in hypertrophic astrocytes at 24 and 72 h **(D-I)**, notably at 24 h **(E)**. Colocalized expression of VEGF with GFAP immunoreactive astrocytes (arrows) and the lumen of blood vessels (asterisks) can be seen in **C**, **F** **I**, and **L**. Immunoreactivity for both VEGF and GFAP is attenuated in the SI + AG group at 72 h, as compared with the SI + S group at corresponding time point **(J-L)**. Scale bar =20.0 μm.

Expression of VEGF was observed in widely distributed branched cells identified as GFAP-expressing astrocytes in the external plexiform layer, mitral cell layer, and internal plexiform layer (Figure 5). In the C group (Figure 5A,B), GFAP and VEGF colocalization was identifiable at moderate levels (Figure 5C). In the SI + S group at 24 and 72 h, both GFAP and VEGF immunoreactivity was markedly enhanced (Figure 5D-I), being most conspicuous at 24 h. The vascular profiles appeared dilated with associated GFAP + VEGF-positive cell processes (Figure 5F,I), notably at 24 h. Both GFAP and VEGF immunofluorescence was moderately attenuated at 72 h in the SI + AG group (Figure 5J-L).

#### Expression of AQP4 and GFAP

**Figure 6:**
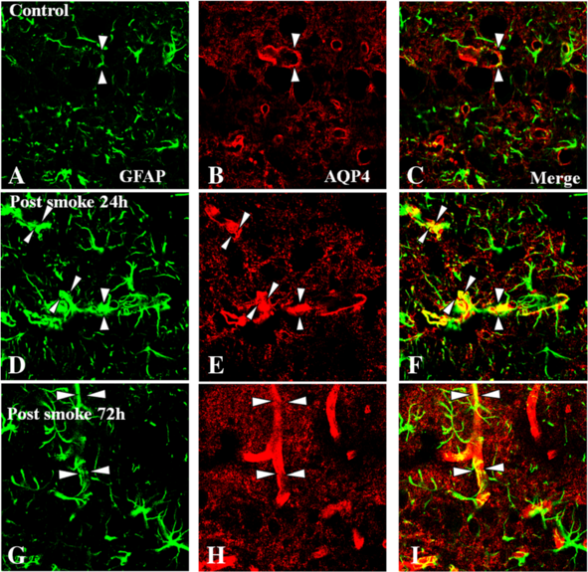
**Immunoexpression of AQP4 (red) and GFAP (green) in the olfactory bulb of the C group (A-C) and the SI + S group at 24 and 72 h (D-I).** In the normal olfactory tissue, AQP4 expression is localized in the astrocytic foot processes (arrows) and blood vessels (arrowheads) **(A-C)**. In the SI + S group, AQP4 expression is increased at different time points (24 and 72 h, **D-I**), being more conspicuous at 24 h **(E)**. Intense AQP4 immunoreactivity is localized in GFAP-labeled astrocyte processes and end-feet and blood vessels, which are well delineated **(C,F,I)**. Aminoguanidine administration did not suppress the immunofluorescence at 72 h (image not shown). Scale bar =20.0 μm.

Expression of AQP4 was ubiquitous in the astrocytic foot processes associated with the blood vessels in the external plexiform layer, mitral cell layer, and internal plexiform layer (Figure 6). Double labeling with GFAP confirmed that the AQP4-positive cells were astrocytes. In the C group, both GFAP and AQP4 immunoreactivity were moderate in the olfactory bulb (Figure 6A,B); GFAP and AQP4 colocalization was only evident on closer examination (Figure 6C). In the SI + S group at 24 and 72 h, the immunoexpression for both markers was drastically enhanced (Figure 6D-I). Strikingly, GFAP positive hypertrophic AQP4-positive astrocytic foot processes impinged on dilated blood vessels, which exhibited colocalization of GFAP immunofluorescence (Figure 6D-I). The AQP4 immunofluorescence, however, was not affected in the SI + AG group at 72 h, as compared with non-treated rats at the same time point (image not shown).

#### Expression of nNOS and NeuN

**Figure 7:**
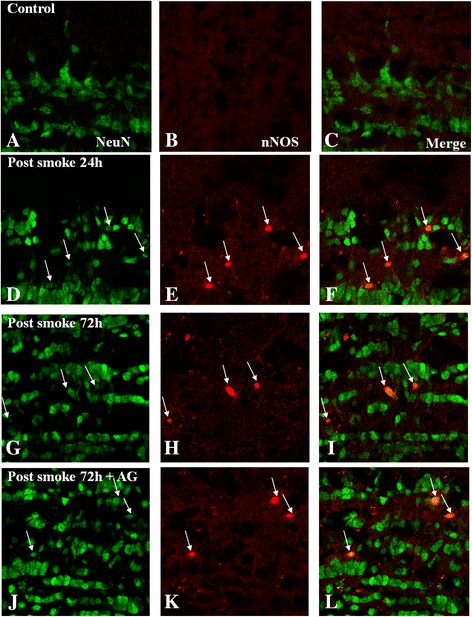
**Immunoexpression of nNOS (red) and NeuN (green) in the olfactory bulb of the C group (A-C), the SI + S group at 24 and 72 h (D-I), and the SI + AG group at 72 h (J-L).** In the normal bulb, nNOS expression is absent **(A-C)**; however, in the SI + S group, it is induced in some neurons at different time points (24 and 72 h, **D-I**); the incidence of labeled cells is higher at 24 h **(E)**. Aminoguanidine administration did not obviously alter the immunofluorescence at 72 h **(J-L)**. Scale bar =20.0 μm.

Expression of nNOS was observed in neurons in the external plexiform layer, mitral cell layer, internal plexiform layer, and granule cell layer (Figure 7). The nNOS-expressing cells were identified as neurons with colocalization with NeuN by double immunofluorescence labeling (Figure 7). In the C group (Figure 7A,B), nNOS and NeuN colocalization could hardly be detected (Figure 7C). In the SI + S group at 24 and 72 h, nNOS intense immunoreactivity was detected in some NeuN-positive neurons (Figure 7D-I) with a higher incidence at 24 h. Intense nNOS immunoreactivity was localized in the nucleus, whose outlined is well defined (Figure 7F,I,L). Aminoguanidine did not obviously suppress the immunofluorescence of nNOS at 72 h (Figure 7J-L).

#### Expression of NKCC1 and GFAP

**Figure 8:**
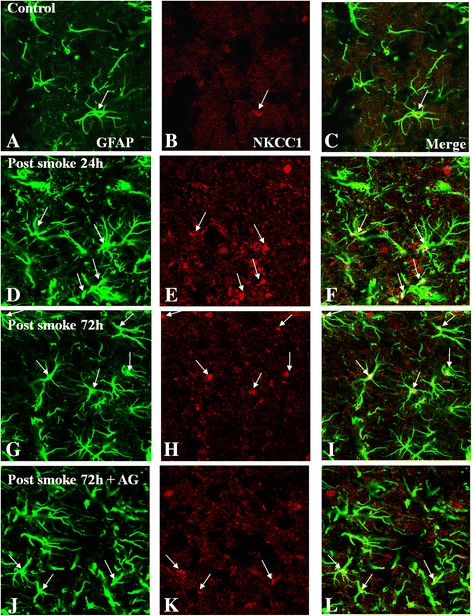
**NKCC1 (red) and GFAP (green) immunopositive astrocytes in the bulb tissue of the control group (A-C), the SI + S group at 24 and 72 h (D-I), and the SI + AG group at 72 h (J-L).** In the C group, only sporadic astrocytes exhibit moderate NKCC1 immunofluorescence. In the SI + S group, it is evidently enhanced in hypertrophic astrocytes at 24 and 72 h **(D-I)**, being more pronounced at 24 h **(E)**. Colocalized expression of NKCC1 with GFAP immunoreactive astrocytes (arrows) can be seen in **C**, **F**, **I** and **L**. Immunoreactivity for both NKCC1 and GFAP is attenuated at 72 h in the SI + AG group, as compared with the SI + S group at 72 h **(J-L)**. Scale bar =20.0 μm.

Expression of NKCC1 was observed in some GFAP-expressing cells in the external plexiform layer, mitral cell layer, and internal plexiform layer (Figure 8). In the C group (Figure 8A,B), moderate NKCC1 immunofluorescence was detected in the soma of occasional astrocytes (Figure 8C). In the SI + S group at 24 and 72 h, both GFAP and NKCC1 immunoreactivity were markedly increased (Figure 8D-I), notably at 24 h. In the SI + AG group at 72 h, both GFAP and NKCC1 immunofluorescence were decreased, as compared with the SI + S group at the same time point (Figure 8J-L).

#### Expression of NKCC1 and NeuN

**Figure 9:**
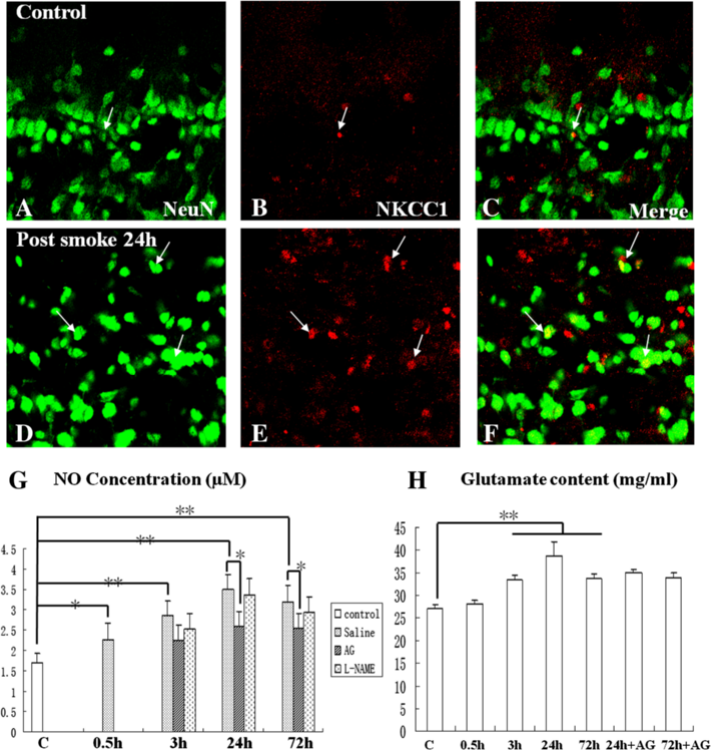
**Immunoexpression of NKCC1 (red) and NeuN (green) in different layers of the olfactory bulb (A-F).** Scale bar =20 μm **(A-F)**. NO content (μM) and glutamate concentration (mg/ml) analysis **(G,H)** in the olfactory bulb of the C group, the SI + S group (0.5, 3, 24, and 72 h) and the SI + AG or SI + L-NAME groups (3, 24, and 72 h) compared with the C group. (* *P* <0.05; ** *P* <0.01). **(A-F)** Colocalized expression of NKCC1 with NeuN immunoreactive neurons (arrows) is detected in the C group. Note that a large number of NeuN-positive neurons are double labeled with NKCC1 **(F)** compared with the C group **(C)**. **(G)** [NO]/μM is significantly increased in the olfactory bulb in SI + S group at 0.5, 3, 24, and 72 h, as compared with the C group; however, [NO] is significantly suppressed in SI + AG group at 24 and 72 h, as compared with saline administration; [NO] is not significantly suppressed at 24 and 72 h in the SI + L-NAME group, as compared with the SI + S group. **(H)** Glutamate concentration is significantly increased in the olfactory bulb in the SI + S group at 3, 24, and 72 h, as compared with that of the C group; it is not significantly suppressed in the SI + AG group at 24 and 72 h, as compared with the SI + S group.

Expression of NKCC1 was localized in some astrocytes, but some NKCC1-expressing cells were identified as the neurons labeled by NeuN (Figure 9A-F). In the C group (Figure 9A,B), NKCC1 immunofluorescence was detected in sporadic neurons (Figure 9C). In the SI + S group at 24 h, the incidence of NKCC1-expressing neurons was increased (Figure 9D-F).

### Aminoguanidine decreased nitric oxide levels in the olfactory bulb after smoke inhalation

The NO levels in the olfactory bulb tissues, as determined by nitrite level, were significantly increased (*P* <0.01) in the SI + S group at 0.5, 3, 24, and 72 h, as compared with the C group (Figure 9G). Aminoguanidine administration (SI + AG) significantly suppressed NO production at 24 and 72 h (*P* <0.05), as compared with the SI + S group (Figure 9G). In the SI + L-NAME group, NO levels remained relatively unchanged at 3, 24, and 72 h, as compared with the SI + S group (Figure 9G).

### Elevated glutamate in olfactory bulb after smoke inhalation

Glutamate levels in the samples of olfactory bulb were significantly increased in the SI + S group at 3, 24, and 72 h (*P* <0.01), peaking at 24 h in comparison with the control levels (Figure 9H). Upon aminoguanidine administration (SI + AG), glutamate levels appeared to decrease at 24 and 72 h, compared with the SI + S group, but this was not significant (Figure 9H).

### RITC extravasation in olfactory bulb after smoke inhalation

**Figure 10:**
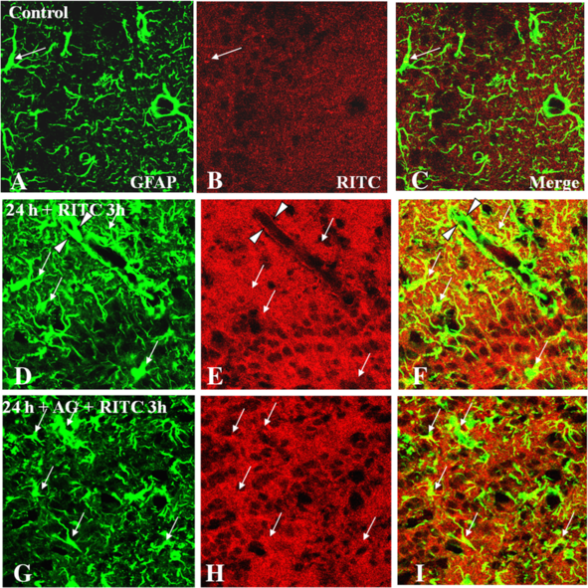
**Extravasation of RITC (red) in the olfactory bulb of the C group (A-C), the SI + S group at 24 h (D-F), and the SI + AG group at 24 h (G-I).** In the C group, the olfactory bulb tissue emits very weak RITC fluorescence **(B,C)**, which is also detected in GFAP-labeled astrocytes (arrow) in **B**. In the SI + S group and at 3 h after RITC administration, RITC fluorescence (asterisks) appears to permeate the neuropil **(D-F)**. In some areas, extravasated RITC superimposes with the blood vessels (arrowhead) **(F,I)**. In the SI + AG group, RITC leakage is moderately reduced, as shown by the attenuated fluorescence **(G-I)**. Scale bar =20.0 μm).

In the control group, RITC extravasation was not evident in the different layers of the olfactory bulb (Figure 10B). RITC was barely detected in GFAP-labeled astrocytes in the C group (Figure 10A-C). In the SI + S group, the olfactory bulb tissue was inundated with RITC dye, which permeated the entire neuropil (Figure 10E). In some areas, extravasated RITC overlapped with the blood vessels, as revealed by GFAP immunofluorescence labeling (Figure 10F,I). The extravasated RITC appeared to be internalized by the astrocytes, as demonstrated by its colocalization with GFAP. In the SI + AG group, RITC leakage was only moderately reduced (Figure 10H).

### Smoke inhalation increased apoptosis in olfactory bulb

**Figure 11:**
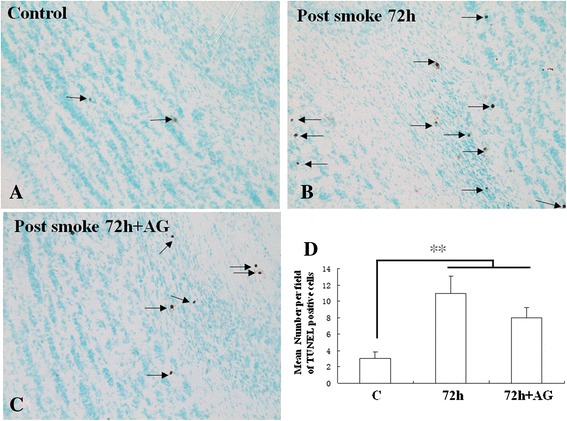
**TUNEL immunopositive cells in the external plexiform layer, mitral cell layer, internal plexiform layer, and granule cell layer in the C group (A), the SI + S group at 72 h (B), and the SI + AG group at 72 h (C).** Smoke inhalation increases the incidence of apoptotic cells (arrows) in the mitral cell layer **(B)** compared with the C group **(A)**. The frequency of apoptotic cells in the olfactory bulb is significantly reduced in the SI + AG group at 72 h, as compared with the SI + S group **(C)**. Scale bar =50 μm. **(D)** Bar graphs show increase in apoptotic cells in the SI + S group at 72 h, as compared with the C group. Apoptotic cells are significantly suppressed in numbers in the SI + AG group at 72 h, as compared with SI + S.

Smoke inhalation-induced apoptosis was observed in the external plexiform layer, mitral cell layer, internal plexiform layer, and granule cell layer (Figure 11). The number of cells undergoing apoptosis as evidenced by TUNEL labeling was markedly increased (*P* <0.01) in the SI + S group at 72 h, as compared with the C group (Figure 11A-D). In the SI + AG group, the number of TUNEL-positive cells was reduced compared with those without drug treatment at 72 h, but the decrease was not statistically significant (Figure 11B-D).

## Discussion

The effect of combustion smoke inhalation on the central nervous system has been reported, but there is a lack of information on its effects on the olfactory bulb. Injury to the central nervous system has been attributed to several factors, such as acute or chronic respiratory distress [22], ischemia, and carbon monoxide intoxication [23,24]. We previously demonstrated that a hypoxic high carbon monoxide environment resulted in drastic systemic and central nervous system changes in an experimental rodent model of combustion smoke inhalation [12,13]. This study aims to determine the effect of smoke inhalation on the olfactory bulb. The present findings suggest that the upregulation of iNOS in response to smoke inhalation plays a major role in olfactory bulb inflammatory pathophysiology after smoke inhalation, with concomitant increases in pro-inflammatory molecules, vascular permeability, and edema.

The pathologic or pathophysiologic changes induced by carbon monoxide or hypoxic exposure to the olfactory bulb remains unclear. A study investigating the effects of smoke on the central nervous system reported that nicotine was the principal pharmacological constituent of tobacco smoke [25]. A hypoxic stimulus to the brain has been associated with the production of large amounts of inflammatory mediators, such as TNF-α and IL-1β [26,27]. In this study, expression levels of TNF-α, IL-1β, and IL-12 were immediately increased in the olfactory bulb at 24 h after smoke inhalation. This suggests the initiation of an acute inflammatory response in the olfactory bulb in response to smoke. There are a number of possible mediators in this reactive response to the smoke inhalation insult.

It has been reported that VEGF plays a role in the inflammatory process by inducing the expression of iNOS and eNOS [28]. Moreover, VEGF can induce NO production and eNOS phosphorylation [29]. VEGF may play a modulating role in the central nervous system by mediating the permeability of the blood-brain barrier. In doing so, the brain may now become more immunocompromised, as it is exposed to blood-borne immune mediators that are normally sequestered away from the brain [30]. In this study, an immediate increase in protein expression of VEGF, iNOS, eNOS, and nNOS was observed at 3 and 24 h after combustion smoke inhalation in the olfactory bulb. The enhanced expression of iNOS was decreased by aminoguanidine administration at 24 and 72 h. In addition, VEGF plays a role in mediating vasodilation and, consequently, the movement of water molecules and large molecular weight proteins from blood vessels into the tissue, resulting in edema [31]. VEGF is overexpressed in reactive astrocytes under pathologic conditions, and has been associated with the breakdown of the blood-brain barrier [30]. The suppression of NO production in endothelial cells has been reported to inhibit endothelial permeability due to VEGF [32]. In this study, we showed that VEGF and NO levels were both increased in the olfactory bulb at 24 and 72 h after smoke inhalation. In particular, VEGF and NO levels were mainly enhanced in the blood vessels and astrocytes. The increase in VEGF expression was associated with an increased in the permeability of the blood vessels in the olfactory bulb, as demonstrated by a leakage of RITC from the periphery into the olfactory bulb tissue at 24 h after smoke inhalation. This suggests that VEGF may mediate vascular permeability. Aminoguanidine treatment drastically decreased the expression levels of VEGF, GFAP, and NO at 24 and 72 h after smoke inhalation, as confirmed by both immunofluorescence and Western blot analysis. Aminoguanidine also decreased RITC intensity in the olfactory bulb, albeit moderately, at 24 h after smoke inhalation, suggesting that aminoguanidine administration can decrease vascular permeability, VEGF expression, and GFAP + astrocyte hypertrophy after smoke inhalation.

Nitric oxide is a small gaseous molecule. Physiologically, nitric oxide plays roles in vasodilation and hence vascular permeability [33,34], neurotransmission and antiplatelet aggregation. Pathologically, nitric oxide may serve to trigger inflammatory pathways, owing to increased upregulation from either neuronal, endothelial or inducible NOS, and may also function as a free radical, contributing to oxidative damage. As in our previous studies, a significant increase in NO production was observed in the olfactory bulb after smoke inhalation in addition to the other parts of the central nervous system investigated [12,13].

To determine the source of NO production in response to smoke inhalation, the selective iNOS inhibitor (aminoguanidine) or nNOS/eNOS constitutive inhibitor (L-NAME) [35] was administered separately. Nitric oxide levels were significantly suppressed with aminoguanidine administration but not with L-NAME administration. This suggests that iNOS is responsible for NO production in response to the smoke challenge and that NO production may mediate the permeability of blood vessels.

AQP4 is a membrane protein that allows for the passage of water through the cell membrane. In particular, AQP4 is found in high concentrations in the perivascular membranes of astrocytes in various mammalian brains [36-38]. AQP4 is also expressed in astrocytic end-feet surrounding endothelial cells lining blood vessels [39]. AQP4 has been implicated in the formation of brain edema due to various injurious insults [36-38]. We have previously reported AQP4 upregulation in the retina and cerebellum of adult rats subjected to smoke inhalation, suggesting its involvement in edema formation of the retina and brain [12,13]. In this study, AQP4 expression was markedly increased in the olfactory bulb after smoke inhalation, especially in the astrocytic foot processes. This further supports the intimate relationship between blood-brain barrier function and the control of water flow by astrocytes. Furthermore, AQP4 has been reported to be strongly expressed in the glomerulus of the olfactory bulb and might play a role in olfactory function [40]. The disruption of olfaction may predispose the individual to neurodegenerative diseases and may also contribute to olfactory problems in dangerous environments, such as in a fire. It is unclear whether the sense of smell is affected by smoke. The animals were observed to have labored breathing immediately after smoke inhalation with recovery to basal rates by 24 h after smoke inhalation. No other behavioral changes were observed. Long-term effects on smell and behavior was also not observed, as animals were sacrificed by 2 w after smoke inhalation.

In this study, RITC was shown to permeate the neuropil in rats subjected to smoke inhalation; furthermore, the tracer extravasation appeared to be acute, as it was evident as early 3 h after its administration. The same phenomenon was also observed in the cerebellum and hippocampus after smoke inhalation. These results suggest that vascular permeability in the olfactory bulb is vulnerable to smoke inhalation and that the integrity of the blood-brain barrier was compromised. One contributing factor to this would be the upregulated AQP4 expression in the olfactory bulb after smoke inhalation.

NKCC1 activates the mitogen-activated protein kinase (MAPK) cascade, which serves to induce edema and brain injury in response to TBI [18]. In a separate study, NKCC1 has also been shown to mediate inflammatory responses where inhibition of NKCC1 prevents the progression of inflammation [41]. In this study, we also found increased NKCC1 protein expression, along with various cytokines and chemokines at 24 and 72 h after smoke inhalation. Immunoexpression of NKCC1 was specifically localized in GFAP + hypertrophic astrocytes as well as NeuN + neurons at 24 and 72 h after smoke inhalation. It has been reported that oxygen-glucose deprivation induced approximately 67% cell death and a four-fold increase in NKCC1+ cortical neurons. The inhibition of NKCC1 resulted in a protection of NKCC1 expressing neurons and astrocytes. These suggest that enhanced NKCC1 activity is linked to ischemic neuronal damage [42]. In our study, a significant increase in apoptosis appeared to coincide with increased NKCC1 protein expression at 72 h after smoke inhalation, thus supporting the notion that both are interrelated.

In another study, exposure to nanoparticle-rich diesel exhaust resulted in glutamate-induced neurotoxicity accompanied by changes in the expression of N-methyl-D-aspartate receptor subunits and related kinase and transcription factor in the mouse olfactory bulb [43]. The present results show that the glutamate levels in olfactory bulb tissue increased at 24 and 72 h after smoke inhalation, suggesting that glutamate homeostasis is disrupted after smoke inhalation and might play a role in altering olfactory function. It is possible that glutamate-induced toxicity might contribute to cell death in the olfactory bulb after smoke inhalation.

## Conclusions

Combustion smoke inhalation upregulates the expression or production of VEGF, GFAP, AQP4, NKCC1, iNOS, eNOS, nNOS, NO, and glutamate in the adult rat olfactory bulb. This was coupled by a rise in inflammatory mediators and an increase in apoptosis, increased blood permeability, and tissue edema. It is suggested that these changes might collectively contribute to olfactory bulb injury after smoke inhalation. It is postulated that hypoxia and high levels of carbon monoxide m cause the changes in the olfactory bulb. Administration of aminoguanidine, an iNOS inhibitor, was found to reduce the production of NO and the extravasation of RITC dye into the olfactory bulb, implicating iNOS as a major contributor in the pathophysiology of olfactory bulb injury after smoke inhalation.

## Abbreviations

ANOVA: analysis of variance
AQP4: aquaporin-4
BSA: bovine serum albumin
C: control
DAPI: 4',6-diamidino-2-phenylindole
ELISA: enzyme-linked immunosorbent assay
eNOS: endothelial nitric oxide synthase
GFAP: glial fibrillary acidic protein
H & E: hematoxylin and eosin
IL-1β: interleukin-1 β
iNOS: inducible nitric oxide synthase
L-NAME: L-NG-nitroarginine methyl ester
MAPK: mitogen-activated protein kinase
NKCC1: Na^+^-K^+^-Cl^−^ cotransporter 1
NOS: nitric oxide synthase
nNOS: neuronal nitric oxide synthase
PBS: phosphate buffered saline
RITC: rhodamine isothiocyanate
SI + AG: smoke inhalation plus aminoguanidine
SI + L-NAME: smoke inhalation plus NG nitro L arginine methyl ester
SI + S: smoke inhalation plus saline
TBS: Tris-buffered saline
TNF-α: tumor necrosis factor-α
TUNEL: terminal deoxynucleotidyl transferase dUTP nick end labeling
VEGF: vascular endothelial growth factor
